# Delphi-based Spanish consensus on the use of long-acting growth hormone in pediatric growth hormone deficiency: recommendations from the ConverGHe Working Group

**DOI:** 10.3389/fendo.2025.1718161

**Published:** 2025-12-04

**Authors:** Lidia Castro-Feijóo, José-Ignacio Labarta-Aizpún, Marta Ramon-Krauel, Diana Primiano, Diego Yeste Fernández, Isabel González Casado

**Affiliations:** 1 Unidad de Endocrinología Pediátrica, Hospital Clínico Universitario de Santiago de Compostela, Instituto de Investigación Sanitaria de Santiago de Compostela, IDIS, Santiago de Compostela, Spain; 2 Unidad de Endocrinología, Servicio de Pediatría, Hospital Universitario Miguel Servet, Instituto Investigación Sanitaria Aragón, Zaragoza, Spain; 3 Servicio de Endocrinología Pediátrica, Hospital Sant Joan de Déu, Institut de Recerca Sant Joan de Déu, Esplugues de Llobregat, Barcelona, Spain; 4 Medical Affairs, Pfizer S.L.U., Madrid, Spain; 5 Servicio de Endocrinología Pediátrica, Hospital Universitari Vall d’Hebron, Universitat Autònoma de Barcelona, Barcelona, Spain; 6 Servicio de Endocrinología Pediátrica, Hospital Universitario La Paz, Madrid, Spain

**Keywords:** long-acting growth hormone, pediatric growth hormone deficiency, recombinant human growth hormone, Delphi consensus, children

## Abstract

**Background:**

Long-acting growth hormone (LAGH) formulations have emerged as an alternative to daily recombinant human growth hormone (rhGH) in pediatric growth hormone deficiency (GHD). Although evidence from meta-analyses and randomized trials supports their efficacy and safety, real-life data are still lacking, and therefore clinical guidance on their implementation remains limited.

**Objective:**

To develop expert-based recommendations for the clinical use of LAGH in pediatric GHD within Spain.

**Methods:**

A two-round Delphi approach was used. Based on a literature review, a scientific committee comprised of pediatric endocrinologists developed a questionnaire structured into three domains: (1) Main benefits of LAGH *vs*. daily rhGH; (2) Candidate profiles for LAGH therapy; and (3) Considerations for initiating LAGH and treatment monitoring. Panelists rated their level of agreement with each questionnaire statement on a 9-point Likert scale. Consensus was defined as ≥66.67% of responses on the same tertile as the median, labelled as disagreement on scores of 1–3, intermediate on scores of 4–6, and agreement on scores 7–9. Statements that did not reach consensus were reformulated and re-evaluated in round 2.

**Results:**

After two rounds, all statements achieved consensus on agreement. Specifically, consensus was reached on 92% of the statements in round 1 and three revised items related to insulin-like growth factor-1 (IGF-1), pharmacokinetics and dose adjustment criteria, as well as the use of LAGH in patients at risk of hypoglycemia, achieved consensus in round 2. The recommendations emphasize the similar efficacy and safety of LAGH compared to rhGH, raising its special interest in cases of poor adherence to daily formulations (e.g. adolescents, injection-related anxiety, multiple comorbid treatments or complex family circumstances), although LAGH should also be considered in other settings in a patient-centered approach. In addition, they provide guidance on key issues related to adequate dose initiation and titration, as well as therapeutic monitoring.

**Conclusion:**

This is the first Delphi consensus to provide national-level guidance on integrating LAGH into pediatric GHD care, offering practical recommendations based on current evidence and national expert opinion. Future long-term real-world experience will contribute to address questions regarding long-term efficacy and safety, and practice-related points such as indication and monitoring.

## 1 Introduction

Growth hormone deficiency (GHD) is a rare condition with an estimated incidence ranging from 1 in 3,000 to 1 in 10,000 live births (1–3). It occurs due to an inadequate secretion of growth hormone (GH), leading to growth-retardation, biochemical, metabolic, and psychological defects if untreated. GHD is typically diagnosed based on growth patterns and confirmed through biochemical testing, including GH stimulation tests, insulin-like growth factor-1 (IGF-1) and insulin-like growth factor-binding protein 3 (IGFBP-3) levels and, when necessary, neuroimaging of the pituitary region and genetic studies. It may be idiopathic, congenital or acquired, and present either as an isolated or as part of combined pituitary hormone deficiencies (4).

The standard of care for pediatric GHD for over three decades has been daily subcutaneous injections of recombinant human GH (rhGH), which have proven to be safe and effective in increasing growth velocity and achieving expected adult height (4, 5). Despite the effectiveness of daily rhGH, the burden of daily injections can negatively impact adherence, particularly in children and adolescents. Studies have shown that up to two-thirds of pediatric patients miss more than one injection per week, a factor strongly associated with suboptimal growth outcomes (6–8). Furthermore, adherence has been shown to decrease from 85% to 74% from the first to the third year of GH replacement, with the greatest decrease in males, adolescents and patients with childhood-onset GHD (9). Challenges to adherence may include needle aversion, inconsistent home routines, or lack of caregiver support.

To address these limitations, long-acting GH (LAGH) formulations have been developed to reduce injection frequency to weekly dosing while maintaining therapeutic efficacy, with non-inferiority demonstrated in pivotal phase III trials when compared to daily rhGH (10–12). Several formulations of LAGH with different pharmacokinetic and pharmacodynamic profile were developed and are currently being studied in children. Those include unmodified rhGH in a depot formulation (Eutropin Plus^®^, LG Life Sciences, approved in South Korea), pegylated rhGH (Jintrolong^®^, GenSci, approved in China), modified rhGH with increased albumin binding (somapacitan, Sogroya^®^, Novo Nordisk), prodrug formulation (lonapegsomatropin, Skytrofa^®^, Ascendis Pharma) and rhGH fusion proteins (somatrogon, NGENLA^®^, Pfizer/OPKO) (13). Of these, only somatrogon and somapacitan included Spanish patients in their respective clinical development programs and have been provided with a national code by the Spanish Agency of Medicines and Medical Devices (AEMPS), with somatrogon being the unique LAGH formulation currently marketed in Spain for the treatment of children and adolescents from 3 years of age with growth disturbance due to insufficient secretion of GH (4). In this regard, it should be emphasized that, while the indications currently approved by regulatory agencies for rhGH treatment also include growth disorders associated with other non-GHD conditions, for the time being the indications for LAGH are limited to GHD (14).

Although treatment with daily rhGH has been shown to be safe and effective in the long-term (5), similar studies to evaluate LAGH are not yet available. Additionally, the unique pharmacokinetic and pharmacodynamic profile of each LAGH requires different dosing and monitoring compared to daily rhGH injections, which may pose an added challenge for the management of these formulations (15, 16). International consensus statements have begun to address some of these gaps (17), but national guidance is needed to align with local regulations, practices, and patient needs. This consensus aims to provide clear guidance on how to integrate LAGH into the routine clinical care of pediatric patients with GHD, based on expert recommendations and supported by current evidence. This will facilitate suitable patient selection, proper treatment initiation and long-term monitoring, while also highlighting current gaps to guide future efforts.

## 2 Methods

### 2.1 Study design and participants

This study comprised a systematic literature review, followed by the development of evidence-based statements, which were then evaluated using a two-round Delphi consensus methodology.

The process involved a scientific committee comprising three advisors and 27 panelists, all pediatric endocrinologists practicing in Spain. Advisors were designated based on their expertise in treating children with daily rhGH and LAGH, as well as their involvement in LAGH publications and clinical trials. As the Delphi methodology is qualitative by nature, the expert committee selected panelists to vote on the statements based on several criteria, including membership of leading scientific societies such as the Spanish Society of Pediatric Endocrinology (SEEP), and having more than 10 years’ experience in managing pediatric GHD.

### 2.2 Systematic literature review

A targeted literature review was conducted using PubMed and Embase, applying both free-text and MeSH terms, aimed at identifying key evidence regarding LAGH and potential controversies. Search terms included growth-hormone deficiency (GHD) therapy, recombinant human growth hormone (rhGH), long-acting growth hormone (LAGH), treatment efficacy and safety, adherence and convenience, quality of life, treatment monitoring, and candidate patient profiles. The search focused on phase II and III clinical trials, regulatory documents, relevant consensus statements, and real-world studies when available, prioritizing those of more recent publication. Additionally, abstracts from international congresses were reviewed to capture emerging data not yet published in journals.

### 2.3 Development of Delphi questionnaire

The advisors reviewed the selected reports from the systematic literature review and drafted a proposal for statements, emphasizing on those topics considered most controversial or relevant. The statements were refined during two in-person meetings held in November 2024 to agree on the final approach. An expert methodologist was present during the meetings to ensure quality and rigor in the elaboration of the statements.

### 2.4 Two-round Delphi process

The Delphi process consisted of two rounds of voting by the expert panel. In round 1, the panel was asked to rate their level of agreement with each statement in the questionnaire on a 9-point Likert scale, from 1 (strongly disagree) to 9 (strongly agree) (18). For this purpose, an in-person meeting was held in January 2025 where voting was conducted anonymously via an interactive platform and comments from the panelists were collected.

The first-round results were discussed by the scientific committee in an online meeting in February 2025. Those statements that did not reach the consensus threshold were modified based on feedback from the expert panel and were either rephrased or split into separate sentences to improve their comprehensibility. In round 2, the updated questionnaire was redistributed, removing all statements that met the consensus threshold in round 1 and including only those that did not, resulting in a splitting of one of these statements into two separate recommendations. Panelists were again asked to rate statements that had not reached consensus using the same criteria described for round 1 via an anonymous online survey to streamline the process.

### 2.5 Statistical analysis

Descriptive statistics were used to summarize the demographic characteristics and the scores obtained for each statement. Consensus was defined as ≥66.67% of responses on the same tertile as the median, labelled as disagreement on scores of 1–3, intermediate on scores of 4–6, and agreement on scores 7–9. The Kolmogorov–Smirnov test was used to evaluate goodness-of-fit of the data to a normal distribution. Internal consistency of the Delphi questionnaire was evaluated with Cronbach’s α (excellent reliability: α≥0.90; good reliability: α = 0.80-0.90; moderate-good reliability: α = 0.70-0.80; moderate-acceptable reliability: α = 0.60-0.70; poor reliability: α = 0.50-0.60; unacceptable reliability: α<0.50) (19). Spearman’s correlation coefficient was used to evaluate the correlation between rounds, by statement and for the entire questionnaire (0-0.25 = slight or no correlation; 0.26-0.50 = weak correlation; 0.51-0.75 = moderate to strong correlation) (20). The increase in the coefficient of variation between the two rounds was analyzed to determine whether a third round was necessary. All the statistical analyses were performed using the Statistical Package for the Social Sciences (SPSS) version 28.0.

## 3 Results

### 3.1 Literature review and Delphi questionnaire

A total of 56 reports were selected for inclusion in the literature review after an assessment of their clinical relevance to the proposed scope. Based on this evidence, the scientific committee finally yielded 37 statements categorized into three major domains: (1) Main benefits of LAGH *vs*. daily rhGH (11 statements); (2) Candidate profiles for LAGH therapy (14 statements); and (3) Considerations for initiating LAGH and treatment monitoring (12 statements). In turn, each domain was divided into several subtopics to facilitate the structuring of the information.

### 3.2 Delphi study

All 27 of the selected panelists completed both rounds of the Delphi questionnaire. A total of 37 statements were initially evaluated across the three domains using a 9-point Likert scale. In round 1, consensus was reached on 34/37 (92%) statements. The remaining items were reconsidered in round 2, after one of the statements was split in two for better understanding, all of which ultimately reached consensus. The median agreement across statements was 88.9% (IQR 7-9). **Supplementary Figure 1** and **Supplementary Figure 2** (**Supplementary Material**) show the results of Likert-scale voting distribution by statement for round 1 and 2, respectively.

The questionnaire had excellent internal consistency (Cronbach α=0.957 [p<0.001] (**Table 1**). The Spearman correlation coefficient for the questionnaire was strong (0.922 [p<0.001]), indicating a high quantitative correlation between the two rounds. The increase in the coefficient of variation between rounds did not exceed 10%, and in absolute terms was less than 1% (0.1870 ± 0.1127 and 0.1781 ± 0.1113 for round 1 and 2, respectively), which justified the decision to forgo a third round.

**Table 1:** Delphi questionnaire reliability.

Domains and statements per domain	Round 1		Round 2	
	Cronbach α	P	Cronbach α	P
Domain 1. Main benefits of LAGH vs. daily rhGH (11/12 statements)	0.897	<0.001	0.835	<0.001
Domain 2. Candidate profiles for LAGH therapy (14 statements)	0.932	<0.001	0.911	<0.001
Domain 3. Considerations at initiation and follow-up of treatment with LAGH (12 statements)	0.748	<0.001	0.836	<0.001
Total (37/38 statements)	0.958	<0.001	0.957	<0.001

LAGH, long-acting growth hormone; rhGH, recombinant human growth hormone.

### 3.3 Consensus statements

#### 3.3.1 Domain 1: main benefits of LAGH *vs*. daily rhGH

This domain included 11 statements that focused on the clinical and perceived advantages of LAGH compared to daily rhGH. Ten of these statements achieved consensus in round 1, while one statement (S11, concerning the interpretation of IGF-1 levels) was revised for round 2. The panel considered the statement too broad, as it combined the pharmacokinetic timing of IGF-1 peaks with whether IGF-1 levels remain within normal ranges. As a result, the item was split into two independent statements, reaching a high degree of agreement for both in round 2 (92.6% and 85.2%, respectively). Regarding the other topics discussed, there was a high degree of agreement that the efficacy of LAGH is similar to that of daily rhGH (88.9%), with no differences observed in the monitoring of laboratory parameters such as metabolic, biochemical, and thyroid function (88.9%), or in bone progression (92.6%), although all panelists unanimously agreed that long-term safety is still unknown. LAGH formulations were also perceived to have a higher incidence of mild injection-site reactions (92.6%) and a higher degree of immunogenicity compared to daily rhGH. However, the latter has no implications on clinical response (96.3%). The panel also reached a high degree of consensus about the lower interference of LAGH in the daily lives of patients and caregivers (96.3%). Although it was also agreed that satisfaction with LAGH is greater and adherence is expected to be higher than with daily rhGH, the percentage of agreement was lower (77.8% and 74.1%, respectively). However, it was emphasized that long-term studies are needed to quantify the impact of LAGH on health-related quality of life (96.3%) (**Table 2**).

**Table 2:** Results of the two-step Delphi process for the statements on domain 1.

Round 1: statements	Percentage IQR [7-9]^a^	Round 2: statements	Percentage IQR [7-9]^a^
Efficacy			
S1. The efficacy of LAGH during treatment is similar and not inferior to the efficacy of daily rhGH.	88.9% Consensus/ Agreement	–	–
Tolerability, side effects and adverse effects			
S2. The tolerability of LAGH treatment is similar to that reported with daily rhGH, although it may present a higher incidence of mild local reactions.	92.6% Consensus/ Agreement	–	–
S3. No significant differences were found in metabolic, biochemical, or thyroid monitoring of LAGH compared to daily rhGH.	88.9% Consensus/ Agreement	–	–
S4. No significant differences were found in bone age progression observed with LAGH compared to daily rhGH.	92.6% Consensus/ Agreement	–	–
S5. Trials have shown a certain degree of immunogenicity with the presence of non-neutralizing anti-LAGH antibodies, but without impact on clinical response.	96.3% Consensus/ Agreement	–	–
S6. The long-term safety of LAGH is unknown, making pharmacovigilance monitoring necessary.	100% Consensus/ Agreement	–	–
Benefits in treatment adherence and convenience			
S7. Although no differences in adherence were found in the trials conducted, it is expected that adherence to LAGH in routine clinical practice would be higher than that of daily rhGH.	74.1% Consensus/ Agreement	–	–
Treatment burden			
S8. LAGH is associated with less interference in daily life compared to daily rhGH for both patients and caregivers.	96.3% Consensus/ Agreement	–	–
S9. Patients and caregivers have greater satisfaction with LAGH compared to daily rhGH.	77.8% Consensus/ Agreement	–	–
Benefits in quality of life and reduction of illness perception			
S10. The impact that LAGH administration has on health-related quality of life requires mid- to long-term, real-world follow-up studies.	96.3% Consensus/ Agreement		
IGF-1 levels			
S11. IGF-1 levels remain within normal range, reaching a peak at 24–48 hours and median level on day 4 after treatment with LAGH.	**63.0%** **Non consensus/** **Agreement**	S11a. IGF-1 levels are dose-dependent, reaching a peak at 24–48 hours, median level on day 4 and trough level on day 6 after LAGH administration.	92.6% Consensus/ Agreement
		S11b. IGF-1 levels on day 4 after LAGH administration generally remain within normal range.	85.2% Consensus/ Agreement

a Consensus was defined as ≥66.67% of responses on the same tertile as the median, labelled as disagreement on scores of 1–3, intermediate on scores of 4–6, and agreement on scores 7–9. Bold serves to highlight the values below the threshold.

IGF-1, insulin-like growth factor 1; IQR: interquartile range; LAGH, long-acting growth hormone; rhGH, recombinant human growth hormone.

#### 3.3.2 Domain 2: candidate profiles for LAGH therapy

Thirteen of the 14 statements in this domain reached consensus in round 1. One statement (S14), related to the non-recommendation of LAGH use in patients with severe GH deficiency and risk of hypoglycemia, did not initially reach consensus and was modified to indicate that caution should be exercised in this case, being accepted in round 2 with a high percentage of agreement (88.9%). There was also a high degree of consensus to not recommend the use of LAGH in specific profiles such as cancer survivors with acquired GHD (88.9%) or patients with GHD associated to other conditions (e.g. mitochondrial disorders, Fanconi anemia, Prader-Willi syndrome, genetic syndromes) until more long-term evidence of its safety profile is generated (92.6%). However, the panel endorsed the use of LAGH in scenarios such as adolescent patients (85.2%), needle phobia or anxiety related to treatment administration (96.3%), poor adherence to daily injections (81.5%), patients with behavioral disorders or neurodiversity (92.6%), and concurrent therapies requiring multiple injections (96.3%). Regarding switching to LAGH, there was consensus that it should be considered whenever requested by the patient or caregiver taking into account the patient’s characteristics (81.5%). Although the agreement was at the threshold limit, the transition to self-administration was perceived as an appropriate moment to inform about the availability of LAGH formulations. Additionally, it was also agreed that proposing the switch to LAGH is not recommended for patients with a good response, tolerance, and satisfaction with daily rhGH (74.1%) (**Table 3**).

**Table 3:** Results of the two-step Delphi process for the statements on domain 2.

Round 1: statements	Percentage IQR [7-9]^a^	Round 2: statements	Percentage IQR [7-9]^a^
Special warnings and precautions for use			
S12. Cancer survivors are not considered a recommended population for initiating LAGH until further evidence is generated regarding its long-term safety.	88.9% Consensus/ Agreement	–	–
S13. Patients with GHD associated to other conditionsb are not considered a recommended population for initiating LAGH until further evidence is generated regarding its long-term safety.	92.6% Consensus/ Agreement	–	–
S14. The use of LAGH is not recommended in patients with severe GHD and risk of hypoglycemia.	**29.6%** **Non consensus/** **Undetermined**	S14. Special caution is recommended when using LAGH in patients with severe GHD and risk of hypoglycemia.	88.9% Consensus/ Agreement
Patient profiles especially indicated: adolescents, low adherence, needle phobia, multiple treatments, special personal and/or family environments			
S15. Adolescents are a group of special interest for recommending treatment with LAGH.	85.2% Consensus/ Agreement	–	–
S16. Patients with low adherencec to daily rhGH are a group of special interest for recommending treatment with LAGH.	81.5% Consensus/ Agreement	–	–
S17. Patients with behavioural disorders or neurodiversity are a group of special interest for recommending treatment with LAGH.	92.6% Consensus/ Agreement	–	–
S18. Patients with needle phobia or anxiety related to treatment administration are a group of special interest for recommending treatment with LAGH.	96.3% Consensus/ Agreement	–	–
S19. Patients with multiple treatments are a group of special interest for recommending treatment with LAGH.	96.3% Consensus/ Agreement	–	–
S20. Patients with family environments or personal circumstances that may make it difficult to adhere to daily treatment are a group of special interest for recommending treatment with LAGH.	92.6% Consensus/ Agreement	–	–
Special considerations regarding to switch			
S21. In patients with good response, tolerance, and satisfaction with daily rhGH treatment, proposing a change of treatment is not recommended.	74.1% Consensus/ Agreement	–	–
S22. Switch to LAGH should be considered based on the request of patients or caregivers, taking into account the patient’s characteristics.	81.5% Consensus/ Agreement	–	–
S23. The transition to self-administration is an appropriate moment to inform about the availability of LAGH formulations.	66.7%Consensus/ Agreement	–	–
S24. In the case of a switch, it is recommended that IGF-1 levels are monitored similarly as for naïve patients.	74.1% Consensus/ Agreement	–	–
Transition to adulthood			
S25. The transition to adulthood is an appropriate moment to inform about the availability of LAGH formulations, considering the indication for adults according to their prescribing information.	81.5% Consensus/ Agreement	–	–

a Consensus was defined as ≥66.67% of responses on the same tertile as the median, labelled as disagreement on scores of 1–3, intermediate on scores of 4–6, and agreement on scores 7–9. Mitochondrial disorders, Fanconi anemia, Prader-Willi syndrome, genetic syndromes.Understood as less than 80%. Bold serves to highlight the values below the threshold.

IGF-1, insulin-like growth factor 1; IQR, interquartile range; LAGH, long-acting growth hormone; rhGH, recombinant human growth hormone.

#### 3.3.3 Domain 3: considerations for initiating LAGH and treatment monitoring

This domain included 12 statements that focused on various considerations for the initiation of treatment with LAGH and proper monitoring of its efficacy and safety. Eleven of these statements achieved consensus in round 1, while one statement (S31, confirmation of IGF-1 level deviation for dose reduction) was revised and accepted in round 2 with a moderate level of agreement (74.1%). Regarding the other topics discussed, there was a high degree of agreement that the decision to initiate treatment with LAGH should be shared between the physician, family and patient (88.9%). All panelists unanimously highlighted the importance of providing therapeutic education on LAGH administration and adequately informing the family about the current state of knowledge about LAGH before initiating treatment. The panel also reached a high degree of consensus that LAGH dose adjustment during follow-up should be individualized based on growth rate, adverse reactions, and serum IGF-1 concentration (96.3%), with dose reassessment initially at 3 months after initiation of treatment and then every 6 months (74.1%). Furthermore, it was also agreed that monitoring of LAGH treatment should include biochemical and hormonal safety profile follow-up 3 months after initiation and every 6–12 months thereafter (81.5%), biannual auxological follow-up (88.9%), and annual bone age monitoring (85.2%). Finally, there was a strong consensus that IGF-1 determination should be performed on day 4 after the last LAGH administration or alternatively by normalization formulas (92.6%), using assays with reliable reference data based on age, sex, and pubertal status (96.3%) (**Table 4**).

**Table 4:** Results of the two-step Delphi process for the statements on domain 3.

Round 1: statements	Percentage IQR [7-9]^a^	Round 2: statements	Percentage IQR [7-9]^a^
Considerations for initiating treatment with LAGH			
S26. Families should be properly informed about current state of knowledge about LAGH before initiating treatment.	100% Consensus/ Agreement	–	–
S27. Decision-making regarding therapeutic alternatives, whether rhGH or LAGH, should be shared between the physician, family, and patient.	88.9% Consensus/ Agreement	–	–
S28. Therapeutic educationb regarding LAGH administration is essential to be offered.	100% Consensus/ Agreement	–	–
LAGH dose adjustment			
S29. LAGH dose adjustment during follow-up should be individualized based on growth rate, adverse reactions, and serum IGF-1 concentrations, without exceeding the therapeutic dose indicated by the prescribing information.	96.3% Consensus/ Agreement	–	–
S30. The LAGH dose should initially be reassessed 3 months after treatment initiation, and subsequently every 6 months.	74.1% Consensus/ Agreement	–	–
S31. If IGF-1 concentrations on day 4 after administration of LAGH dose are greater than 2 SDS, this should be reconfirmed with a new determination in less than 3 months before dose reduction.	**40.7%** **Non consensus/** **Undetermined**	S31. If IGF-1 concentrations on day 4 after administration of LAGH dose are greater than 2 SDS, this should be confirmed with a new determination before reducing the dose.	74.1% Consensus/ Agreement
S32. If IGF-1 concentrations on day 4 after administration of LAGH dose are confirmed to be greater than 2 SDS, the dose will be reduced according to the prescribing information.	88.9% Consensus/ Agreement	–	–
LAGH monitoring			
S33. Auxological monitoring should be performed every 6 months after initiating treatment with LAGH.	88.9% Consensus/ Agreement	–	–
S34. Laboratory test follow-up should be performed at 3 months and subsequently every 6–12 months after initiating treatment with LAGH.	81.5% Consensus/ Agreement	–	–
S35. Bone age should be monitored annually after initiating LAGH treatment.	85.2% Consensus/ Agreement	–	–
S36. IGF-1 determination should be performed on day 4 after the last dose of LAGH administration, or alternatively, normalization formulae should be used.	92.6% Consensus/ Agreement	–	–
S37. IGF-1 measurement should be performed using an assay with reliable reference data, with ranges based on age, gender, and pubertal status.	96.3% Consensus/ Agreement	–	–

a Consensus was defined as ≥66.67% of responses on the same tertile as the median, labelled as disagreement on scores of 1–3, intermediate on scores of 4–6, and agreement on scores 7–9. Missed doses, frequency, rotation, and risk of lipodystrophy. Bold serves to highlight the values below the threshold.

IGF-1, insulin-like growth factor 1; IQR, interquartile range; LAGH, long-acting growth hormone; rhGH, recombinant human growth hormone; SDS, standard deviation score.

## 4 Discussion

This is the first Delphi study regarding the use of LAGH in Spain with the aim of providing guidance on its integration into clinical practice. This issue has been addressed in several recent studies, such as the international consensus published by Maniatis et al., 2025 (17), and the recommendations made by Linglart et al., 2024 (21) which are applicable to the current management of LAGH formulations in France. Our consensus is closely aligned with the conclusions drawn in these studies, further strengthened using a more rigorous, systematized working method, such as the Delphi approach. The study panel was able to reach a high degree of consensus among leading pediatric endocrinologists in Spain on relevant aspects of the clinical use of LAGH formulations in children with GHD, with a particular focus on its potential benefits compared with daily rhGH, main considerations for initiating therapy and treatment monitoring, and patient profiles that may benefit most from a weekly dosing regimen. Nevertheless, this Delphi process also revealed uncertain issues, particularly regarding the lack of long-term data to enhance current knowledge of LAGH, and the need to improve clinicians’ understanding of its unique pharmacokinetic characteristics to enable appropriate dose adjustment and IGF-1 monitoring in both naïve patients and those switching from daily rhGH.

### 4.1 Domain 1: main benefits of LAGH *vs*. daily rhGH

The high degree of consensus reached by the panel reveals that LAGH formulations are perceived as a novel therapeutic option with similar efficacy to daily rhGH in terms of growth velocity, height standard deviation score (SDS), and bone age progression, which is widely described in several published systematic reviews, meta-analyses, and phase II and III pivotal trials (10–12, 22–30). However, it is emphasized that, while the evidence is clear in the short term, data on final adult height outcomes remain limited, making longer-term follow-up necessary. Similarly, the panel’s perception of the safety profile of LAGH is consistent with currently available data, which have shown a similar incidence of treatment-emergent adverse events compared with daily rhGH, in most cases mild or moderate in severity (10, 12, 22–28). Nevertheless, the available clinical trials to date have revealed some differences in tolerability and safety profiles between different LAGH formulations, which explains some of the clarifications included in the consensus statements. On the one hand, while the levels of non-neutralizing anti-drug antibodies observed for somapacitan and lonapegsomatropin were similar to those reported for rhGH (11, 12), an increase was observed with somatrogon (10). However, as confirmed by the published evidence, it should be emphasized that this has no implications on clinical outcomes (10, 12, 29). On the other hand, injection site pain was reported infrequently in trials of somapacitan, variably in lonapegsomatropin trials and at a high frequency in somatrogon trials, however mostly mild to moderate (10), which can probably be explained by factors such as the volume of each injection, preservatives in buffer solution, needle size, or other needle features (23). Regarding local reactions, it is also hypothesized that the higher incidence reported with somatrogon compared to other LAGH formulations may be due to more thorough recording than in other trials (10). Overall, long-term safety remains an important unknown due to the limited duration of follow-up with LAGH compared to daily rhGH (26), emphasizing the importance for future studies and pharmacovigilance monitoring. GH registries are valuable data sources to evaluate efficacy and safety of GH treatment. Long-term phase IV LAGH studies, including real-world practice, will improve understanding and are needed to demonstrate these benefits and the cost-effectiveness of LAGH. GloBE-Reg (Global Registry For Novel Therapies In Rare Bone & Endocrine Conditions) is an international registry project that focuses on effectiveness and safety of any GH treatment, including LAGH. Pfizer is participating in this international registry through Pfizer Registry of Outcomes in Growth hormone RESearch (PROGRES) as a phase IV substudy of daily rhGH and LAGH. Similarly, other industries are also participating such as the REAL10 study of somapacitan in children with GH deficiency. Sharing data in a unique international registry will increase the power of the information obtained and will allow the development of evidence-based information (31, 32).

Regarding adherence to LAGH, current evidence shows no difference compared to daily rhGH, most likely because the studies were not designed to assess this endpoint (10, 28, 29). Nonetheless, although the level of consensus was moderate for this statement, it is hypothesized that reducing the frequency of injections may improve adherence to treatment. In this regard, it is also emphasized that devices which allow for certification and monitoring of adherence are desirable, as they are likely to have a positive impact on therapeutic compliance (33, 34). The available data on the impact of LAGH on health-related quality of life is also at present limited (35), which aligns with the panel’s stance. In this regard, doubts have been expressed as to whether future evidence will be able to provide any conclusive information, primarily because of the way in which it is currently defined in questionnaires and the difficulty of objectively assessing this issue in real-life clinical practice. However, the available evidence does support that LAGH reduces the treatment burden on patients’ and caregivers’ lives (12, 36–39), as reflected by the high level of agreement on these items.

Besides, the unique pharmacokinetic profile of LAGH seems to explain the initial lack of consensus and debate regarding IGF-1 levels. Again, the published evidence shows differences between formulations, documenting an increase in IGF-1 levels with LAGH compared with daily rhGH (10–12, 24–26). However, it is emphasized that the percentage of patients who may present a transient elevation during the initial 24–48 hours after administration above the normal range of 2 SDS is small (40).

### 4.2 Domain 2: candidate profiles for LAGH therapy

Concerning candidate profiles for LAGH therapy, there is no literature directly supporting the use of LAGH in specific circumstances; rather, consensus recommendations are based primarily on indirect evidence (17).

The high degree of consensus reached by the panel reveals that LAGH formulations are perceived as a new therapeutic option especially recommended for patients with poor adherence to daily rhGH, such as adolescent patients in particular, or those with specific situations that may complicate compliance with the therapeutic regimen (e.g. patients with needle phobia or injection-related anxiety, patients with behavioral disorders or neurodiversity, patients on multiple treatments, or patients with special family environments and/or personal circumstances). As described in the literature, adolescents represent a group with particularly low adherence to daily rhGH (41–43). Besides, considering that treatment non-adherence is a complex and multifactorial problem influenced by socio-family factors, injection problems, or treatment fatigue, among others (44–48), it is expected that, due to its dosage characteristics, real-life adherence to LAGH will improve when compared with daily administration formulations. In this regard, several published studies support a lower treatment burden, a better patient and caregiver experience, less interference with daily life, and higher scores regarding therapeutic adherence with LAGH (37, 39). Therefore, its indication for GHD should be always considered on an individual basis, if no contraindications are present. Nevertheless, when it comes to patients receiving multiple treatments, it is also important to carefully assess LAGH treatment, taking patient safety into account based on their underlying pathology.

There was also a high level of agreement to not recommend LAGH treatment for certain special populations, such as cancer survivors or patients with GHD associated with other conditions (e.g., mitochondrial diseases, Fanconi anemia, Prader-Willi syndrome, and other genetic syndromes). Current evidence does not support an association between daily rhGH replacement therapy and primary tumor or cancer recurrence in GHD children (5, 49–51). This information is still unknown for LAGH formulations, emphasizing the need for long-term surveillance into safety in this regard. Nonetheless, the use of LAGH in toddler patients with severe GHD has been subject of discussion due to the high risk of hypoglycemia, particularly given the lower GH levels in the days prior to the next dose. The agreement ultimately reached by the panel concerning this item suggests special caution when considering the use of LAGH in this profile without age specification, assuming that the treatment will be prescribed according to the indications approved by the medical agencies. However, the need to perform blood glucose monitoring is emphasized in severe GHD with risk of hypoglycemia, especially in the 1–2 days prior to each injection (21).

Regarding switching from daily rhGH to LAGH, the panel favored a patient-centered approach. While switching is not recommended in patients with good response, tolerance and satisfaction with daily rhGH treatment, there is a high degree of agreement that LAGH should be considered under an individualized approach if requested by the patient or caregiver, acknowledging the importance of patient preference in decision-making. However, it is striking that the agreement reached regarding the appropriateness of the transition to self-administration to inform about the availability of LAGH formulations is moderate, whereas the transition to adulthood is widely considered to be appropriate. In this regard, it should be noted that somapacitan is already indicated for the treatment of GHD in adults, while somatrogon and lonapegsomatropin are currently restricted to pediatric use. Whenever it occurs, it is recommended that IGF-1 levels are monitored similarly as for naïve patients, bearing in mind that deviations greater than 2 SDS may be more frequent after the switch (52).

### 4.3 Domain 3: considerations for initiating LAGH and treatment monitoring

Regarding considerations prior to initiating treatment, the available evidence supports the fact that, given the different pharmacological characteristics and the lack of long-term experience with these new formulations, LAGH should only be prescribed after the patient and/or their caregivers have been fully informed (53). This is consistent with the panel’s perception, which unanimously agreed on the need to adequately inform the family about the current state of knowledge about LAGH. Similarly, therapeutic education is widely perceived as essential for maximizing the benefits of LAGH therapy. In this regard, the importance of avoiding the erroneous assumption that LAGH does not require injection site rotation due to its lower dosing frequency is emphasized. As stated in the prescribing information for the available formulations, the injection site should be rotated with each administration to avoid the risk of lipodystrophy (54–56). The importance of therapeutic education on other critical aspects, such as the time window for administering a missed dose, is also underscored. This is particularly relevant given that the pharmacokinetics of different LAGH formulations lead to varying timeframes (3 days for somatrogon and somapacitan, and -2/+2 days for lonapegsomatropin) (17). In line with the available evidence, which recognizes the growing interest in involving patients in therapeutic decision-making (39), the panel also acknowledges a high level of consensus on the importance of shared decision-making between physicians, caregivers and patients. However, it should be noted that access to available options may differ between hospitals or regions.

On the other hand, although the starting dose is established by the prescribing information for each product—0.66 mg/kg/week for somatrogon, 0.24 mg/kg/week for lonapegsomatropin and 0.16 mg/kg/week for somapacitan—issues such as dose adjustment during treatment or the management of a suboptimal response remain topics of debate (57). It should be taken into consideration that direct dose comparisons between different formulations, whether between rhGH and LAGH or between available LAGH formulations, are not adequate. In this regard, the panel highly agreed that LAGH dosing during treatment should be individualized based on growth velocity, adverse effects, and serum IGF-1 levels, without exceeding the therapeutic dose approved by the medical agencies. While weight is undoubtedly a factor to consider when determining the initial dose, it does not appear to be as relevant in titration. This suggests that future knowledge may reveal that the dose does not necessarily need to be increased if the patient adequately meets the other criteria at the follow-up visit. However, it is strongly emphasized that its use will always be carried out under the conditions approved by the medical agencies.

Regarding response monitoring, the clinical trials for LAGH included dose adjustments beginning at 3 months and then every 6 months based on efficacy and safety (10–12, 26). However, this statement had a slightly lower consensus, likely reflecting clinical uncertainty about optimal dosing intervals. Furthermore, the frequency of confirming possible deviations in IGF-1 levels above 2 SDS was a topic of discussion, but a moderate consensus was ultimately reached on the proposed statement. It is emphasized that, beyond confirming possible deviations through laboratory results, active investigation should be conducted into whether there is a plausible factor that could explain them. Finally, while the prescribing information for LAGH establish an evaluation of efficacy and safety at 6- to 12-month intervals (54–56), this consensus provides more detailed and agreed-upon follow-up guidelines with a high degree of agreement. Additionally, considering the unique pharmacokinetics of LAGH and the importance of IGF-1 measurement in response monitoring, it is finally agreed that this determination must be performed on day 4 (96 hours) after administration, representing the mean IGF-1 value, ideally through reliable assays, adjusted by pubertal status, sex, race. This differs slightly for lonapegsomatropin, as its monitoring must be performed 108 hours after administration (56, 58). Alternatively, normalization formulae, which can be used to allow adjustments to predict mean IGF-1 and IGF-1 SDS values depending on time after dose, has been created to enable physicians to monitor the treatment (59–61). However, the need to generate more long-term evidence to understand the relationship between estimated peak and estimated average IGF-1 levels at steady state, and its influence on the short- and long-term safety and efficacy of LAGH therapy, is also emphasized.

### 4.4 Limitations and future work

Although the scope of the panel was limited to Spain, this consensus was strengthened by full panel retention across rounds and representation from experienced pediatric endocrinologists from across the country. By combining expert clinical assessment and a targeted literature review, the process yielded practical recommendations to guide national implementation, while remaining adaptable to the evolving international landscape. Although the panel did not include patients or caregivers, literature on patient preferences and treatment burden informed related recommendations, and future updates should incorporate direct patient input where feasible.

Future work should focus on validating these recommendations in real-world settings, particularly regarding adherence, patient-reported outcomes, pharmacoeconomics and cost-effectiveness, the effect on other parameters such as body composition, and safety in underrepresented groups. Continued efforts to harmonize IGF-1 monitoring protocols, refine dose optimization strategies, and generate clinical experience will be essential for the consistent use of LAGH across diverse clinical settings.

## 5 Conclusion

This is the first Delphi consensus that provides expert-based recommendations for the use of long-acting growth hormone (LAGH) in pediatric growth hormone deficiency, based on current evidence and clinical use in Spain. Most statements reached a strong agreement, while areas of uncertainty, mainly related to IGF-1 monitoring and dose adjustment, suggest an evolving clinical practice. The recommendations offer practical guidance for identifying patients who are most likely to benefit from LAGH, particularly those with adherence challenges. Future long-term real-world experience will help to address questions regarding long-term efficacy and safety and practice-related points such as indication and monitoring. Recommendations are intended to be used to support national implementation while remaining adaptable to future international guidelines.

## Data Availability

The original contributions presented in the study are included in the article/**Supplementary Material**. Further inquiries can be directed to the corresponding authors.
