# Supplementary material for: Delphi-based Spanish consensus on the use of long-acting growth hormone in pediatric growth hormone deficiency: recommendations from the ConverGHe Working Group
Source: Front Endocrinol (Lausanne). 2025 Dec 4;16:1718161. doi: 10.3389/fendo.2025.1718161 (PMC12711535; doi:10.3389/fendo.2025.1718161)
Supplement: Supplementary file 1 [file DataSheet1.docx]

**Supplementary Figure 1.** Round 1 Likert-scale voting distribution by statement.


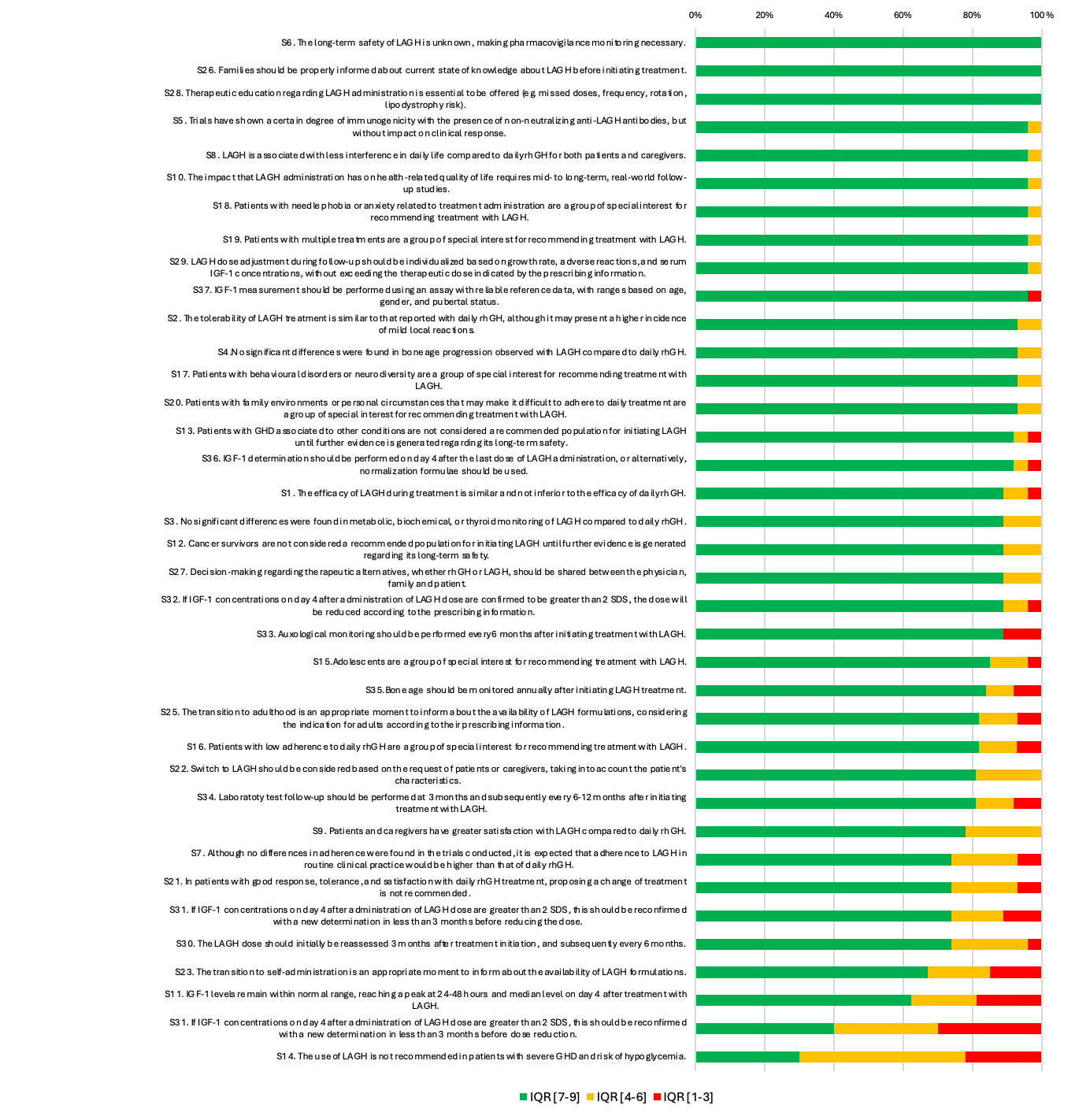


Each bar represents the percentage of panelists whose ratings fell within the three agreement tertiles: agreement (scores 7-9, green), intermediate (scores 4-6, yellow), and disagreement (scores 1-3, red). The black dotted line marks the 66.7% consensus threshold.

**Figure 2.** Round 2 Likert-scale voting distribution by statement.

**IGF-1:** insulin-like growth factor 1; **IQR:** interquartile range; **LAGH:** long-acting growth hormone; **rhGH:** recombinant human growth hormone; **SDS:** standard deviation score.
